# Distinguishing Anuran species by high‐resolution melting analysis of the COI barcode (COI‐HRM)

**DOI:** 10.1002/ece3.5808

**Published:** 2019-11-06

**Authors:** Steven Everman, Shiao Y. Wang

**Affiliations:** ^1^ Department of Biological Sciences The University of Southern Mississippi Long Beach MS USA

**Keywords:** COI, DGGE, high‐resolution melting analysis, HRM, species identification

## Abstract

Taxonomic identification can be difficult when two or more species appear morphologically similar. DNA barcoding based on the sequence of the mitochondrial cytochrome c oxidase 1 gene (COI) is now widely used in identifying animal species. High‐resolution melting analysis (HRM) provides an alternative method for detecting sequence variations among amplicons without having to perform DNA sequencing. The purpose of this study was to determine whether HRM of the COI barcode can be used to distinguish animal species. Using anurans as a model, we found distinct COI melting profiles among three congeners of both *Lithobates* spp. and *Hyla* spp. Sequence variations within species shifted the melting temperature of one or more melting domains slightly but do not affect the distinctness of the melting profiles for each species. An NMDS ordination plot comparing melting peak profiles among eight Anuran species showed overlapping profiles for *Lithobates sphenocephala* and *Gastrophryne carolinensis*. The COI amplicon for both species contained two melting domains with melting temperatures that were similar between the two species. The two species belong to two different families, highlighting the fact that COI melting profiles do not reveal phylogenetic relationships but simply reflect DNA sequence differences among stretches of DNA within amplicons. This study suggests that high‐resolution melting analysis of COI barcodes (COI‐HRM) may be useful as a simple and rapid method to distinguish animal species that appear morphologically similar.

## Graphical abstract

Taxonomic identification can be difficult when two or more species appear morphologically similar. DNA barcoding based on the mitochondrial cytochrome c oxidase 1 gene (COI) is widely used in identifying animal species. High‐resolution melting analysis of COI barcodes (COI‐HRM) provides a simple and rapid method to identify or distinguish animal species without the need to sequence DNA.

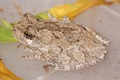

## 1 INTRODUCTION

Taxonomic identification can be difficult when two or more species appear morphologically similar. For example, the frogs *Hyla versicolor* and *Hyla chrysoscelis* share breeding ponds (Harding, 1997) but appear identical and can only be differentiated by their mating calls and karyotype (Collins & Conant, 1998; Jaslow & Vogt, 1977). Among five species of *Radix* snails, intraspecific variability in shell morphology and anatomy overlap among species (Glöer, 2002). For many species, the difficulty is more acute for early life stages such as embryos and juveniles when definitive morphological characteristics are not yet established. DNA‐based species identification, however, is extremely reliable. One widely used approach is DNA barcoding using a region of the mitochondrial cytochrome oxidase subunit I (COI) gene for animal species (Hebert, Cywinska, & Ball, 2003). The gene is PCR‐amplified, sequenced, and then compared to a sequence database for species identification.

An alternative to DNA sequencing for detecting variations among PCR amplicon sequences is high‐resolution melting analysis (HRM). During HRM, the fluorescence of PCR amplicons in the presence of a dsDNA saturating dye is continuously monitored as temperature increases at predetermined increments. Because there is a loss in fluorescence when double‐stranded DNA denatures and the temperature at which DNA melts is sequence‐dependent, unique amplicons produce a unique melting curve. The first derivative of each melting curve can then be plotted against temperature to produce unique melting peak profiles that may be useful for species identification.

The effectiveness of HRM for distinguishing related species is unclear. Of concern is the effect of amplicon size. With primers LCO1490 and HC02198, frequently used to DNA barcode animals, a 658‐bp region of interest within the COI gene is amplified (Folmer, Black, Hoeh, Lutz, & Vrijenhoek, 1994). For screening single‐nucleotide polymorphism, sensitivity and specificity is greater for PCR products of 300 bp or less compared to larger products (Reed & Wittwer, 2004). However, large PCR products can produce melting peak profiles with multiple melting peaks (Lilliebridge, Tong, Giffard, & Holt, 2011; Rasmussen, Saint, & Monis, 2007) that aid in differentiating sequence variants. Indeed, detection of virus serotypes using HRM analysis of a ~600 bp PCR product has been reported (Steer, Kirkpatrick, O'Rourke, & Noormohammadi, 2009).

The goal of the present study was to test the usefulness of high‐resolution melting analysis of the COI barcode (COI‐HRM) to distinguish species using anurans as a model. The sequence of the COI gene has been shown to be effective for identifying anurans to species (Perl et al., 2014; Smith, Poyarkov, & Hebert, 2008; Vences, Thomas, Bonett, & Vieites, 2005). Using DNA collected from eight different species in Hattiesburg, Mississippi, we found that PCR‐HRM of the COI barcode produced melting peak profiles that were highly reproducible and useful for distinguishing species.

## 2 MATERIALS AND METHODS

### 2.1 Tissue collection/DNA extraction

Tissue samples from three adult frogs of each species (Table 1) were collected from a freshwater pond in Hattiesburg, Mississippi, USA (31.20 N 89.22 W). Total DNA was extracted using the DNeasy Tissue Kit (Qiagen) following the manufacturer's protocol. Extracted DNA was diluted using UV‐sterilized DNase/RNase‐free water to a concentration of 10 ng/μl. Tissue sample collection was approved by the Mississippi Department of Wildlife, Fisheries, and Parks (Permit No. 0623151).

**Table 1:** Anuran taxa used in the study

Family	Species
Ranidae	*Lithobates catesbeianus*, *Lithobates clamitans*, *Lithobates sphenocespala*
Hylidae	*Acris gryllus*, *Hyla cinerea*, *Hyla gratiosa*, *Hyla versicolor*
Microhylidae	*Gastrophryne carolinensis*

### 2.2 PCR/HRM

PCR and HRM, using the Rotor‐Gene 6000 (Corbett Life Sciences, now Qiagen), was performed in a 25 μl reaction volume containing 12.5 μl of EconoTaq PLUS 2X Master Mix (Lucigen), 5 μl of 10 ng/μl extracted DNA, 2 μl each of 5 μM forward and reverse primers Chmf4 and Chmr4 described by Che et al. (2012), 1.75 μl of DNase/RNase‐free water, 1.25 μl of 20x EvaGreen (Biotium), and 0.5 μl of 25 mM MgCl_2_ to adjust the final magnesium concentration to 2 mM. The utility of the primers has been confirmed by Chambers and Hebert (2016) in a comprehensive study of North American amphibians.

Initial melting took place at 95°C for 5 min followed by 35 cycles of melting at 94°C for 1 min, annealing at 46°C for 1 min, extension at 72°C for 1 min, and a final extension at 72°C for 10 min. High‐resolution melting analysis was performed immediately after completion of PCR. Sample fluorescence was acquired from 65°C to 95°C at 0.2°C increments, five seconds after each temperature increase had been reached. To better visualize the effect of temperature on DNA melting and the possible presence of multiple melting domains, first derivatives of the change in sample fluorescence over time (−dF/dT) were calculated using the Rotor‐Gene 6000 Series Software (version 1.7) at each 0.2°C increment and plotted against temperature.

### 2.3 PERMANOVA/NMDS

To compare the DNA melting profiles statistically, the relative first derivative of sample fluorescence at each temperature increment between 75°C and 90°C was used to construct dissimilarity matrices based on the Euclidean distance metric. Permutation analysis of variance (PERMANOVA) (Anderson, 2001) with 10,000 permutations was used to test for significant differences among the melting peak profiles of members of both the genus *Lithobates* and *Hyla*. PERMANOVAs were performed using the adonis function of the vegan package (Oksanen et al., 2015) in R. *p*‐values < .05 were considered significant. Nonmetric multidimensional scaling (NMDS), retaining two dimensions, was performed using the metaMDS function of the vegan package in R. Confidence ellipses were drawn at the 50% level to aid in visualizing clusters using the car package (Fox & Weisberg, 2011) in R.

### 2.4 DNA sequencing

PCR products were sequenced commercially (Eurofins MWG Operon LLC) using the Sanger sequencing. Unincorporated nucleotides and primers remaining in PCR products were digested prior to sequencing using ExoSAP‐IT (Affymetrix) according to the manufacturer's protocol. PCR products were sequenced in both directions with primers used during PCR. Primer sequences were removed, and the number of nucleotide mismatches was calculated using the ape package (Paradis & Schliep, 2018) in R (version 3.3.3). Sequences were deposited into GenBank under accession numbers (KT388385–KT388408).

## 3 RESULTS

**Figure 1:**
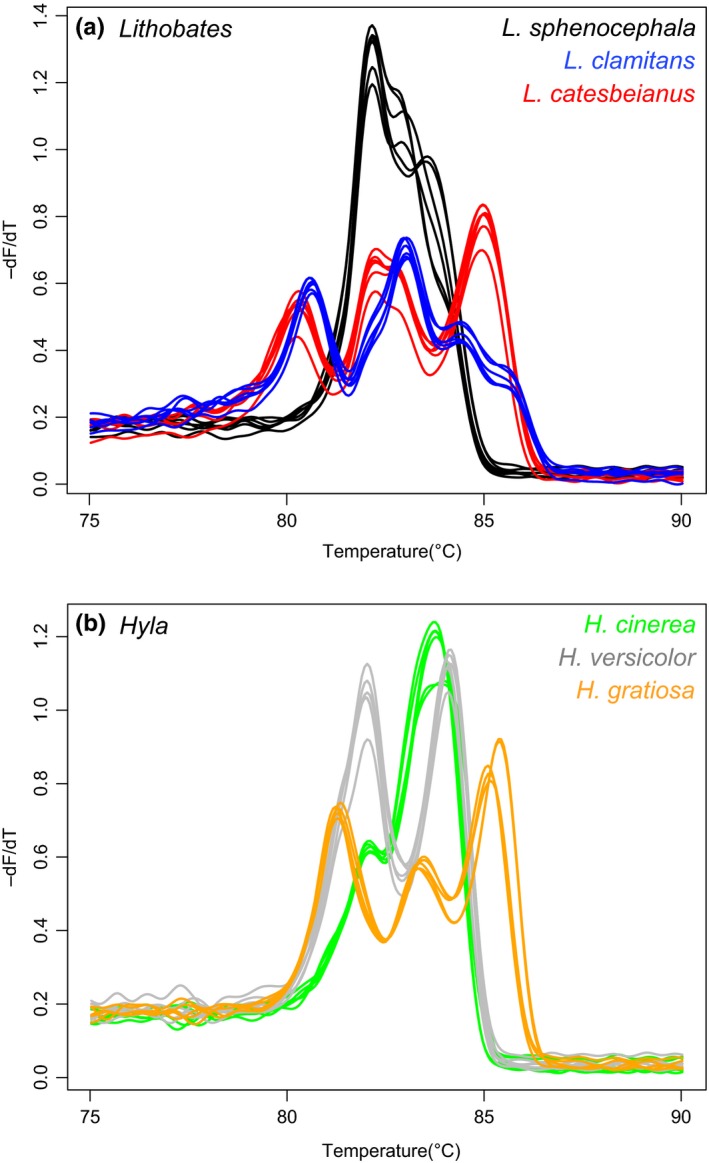
COI barcode melting peak profiles of conspecific Anurans. (a) *Lithobates* species. (b) *Hyla* species. *N* = 3 with technical replicates shown

A single amplicon, 706 bp in length, was amplified from each species collected (data not shown). The amplicons contained up to three melting domains that produced melting profiles that can be used to clearly distinguish congeneric *Lithobates* (Figure 1a) and *Hyla* species (Figure 1b). The melting peak profiles were significantly different among *Lithobates* species (*df* = 2, pseudo‐*F* = 92.24 and *p* = .0038) and among *Hyla* species (*df* = 2, pseudo‐*F* = 52.82 and *p* = .0031).

**Figure 2:**
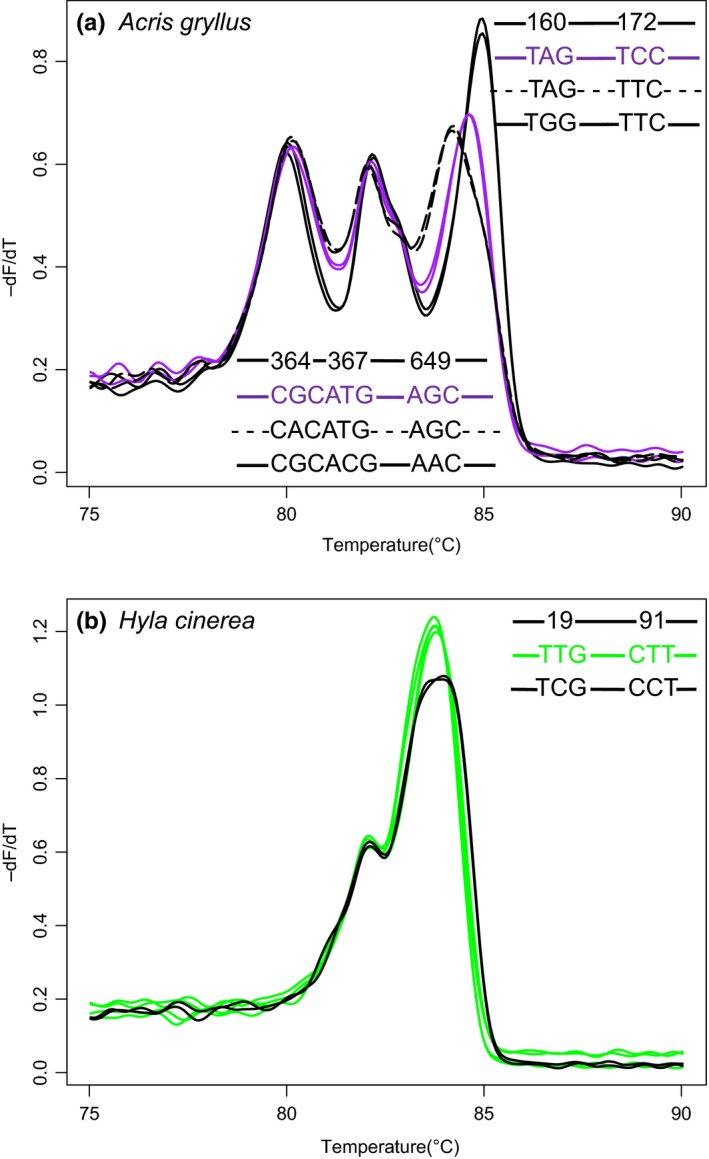
Effect of sequence variation within the COI barcode on melting peak profiles within Anuran species. (a) *Acris gryllus*. (b) *Hyla cinerea*. Numbers represent the position within the amplicon where sequence variation was observed. *N* = 3 with technical replicates shown

The sequence of the COI barcode was identical among conspecifics for two (*Lithobates catesbeianus* and *Lithobates clamitans*) of the eight Anuran species (data not shown). Sequence variations in the six other species shifted the melting temperature of some peaks but do not affect the utility of HRM analysis for distinguishing species. For example, sequence differences among *Lithobates sphenocephala* individuals produced a melting temperature difference of approximately 1.5°C for one of the domains but the amplicon melting profile remains distinct compared to those of two congeners (Figure 1a). The degree of shift seems to correlate with the amount of sequence variation. For example, the shift in melting temperature of one of the melting domains for *Acris gryllus,* with variation between two and four positions, was approximately 1°C whereas for *Hyla cinerea*, with variation at only two positions, the shift was approximately 0.5°C (Figure 2a,b).

**Figure 3:**
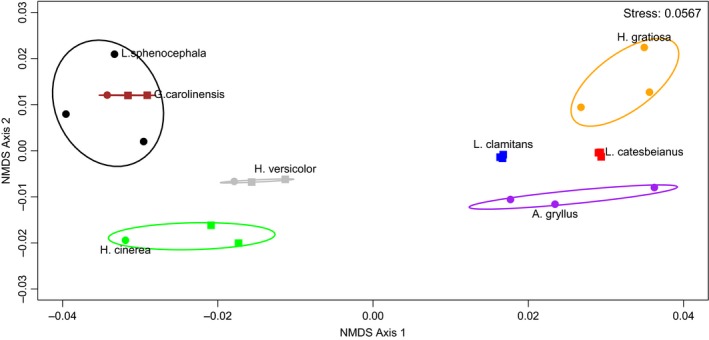
Nonmetric multidimensional scaling ordination plot, using Euclidean distance as the distance metric, summarizing the difference in melting peak profiles among the eight anuran species. Euclidean distance was calculated after technical duplicates were averaged and the relative first derivative values of fluorescence (−dF/dT) were obtained for each specimen. Squares denote sequence identity within the species

**Figure 4:**
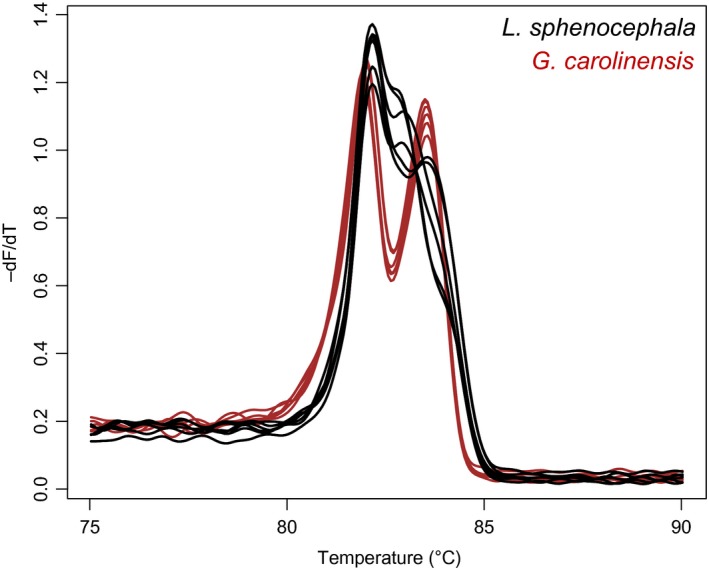
COI melting peak profiles of *Lithobates sphenocespala* and *Gastrophryne carolinensis*. *N* = 3 with technical replicates shown

An NMDS ordination plot (Stress: 0.0567) summarizing the differences in melting peak profiles among the eight Anuran species revealed only six distinct clusters. Clusters containing *L. sphenocephala* and *Gastrophryne carolinensis* specimens overlapped (Figure 3). The amplicon for each species has two melting domains with similar melting temperatures but the melting profiles appear distinguishable due to a distinct valley between melting peaks for *G. carolinensis* (Figure 4).

## 4 DISCUSSION

Accurate species identification is essential in fields such as community ecology and conservation biology. For some animals, the task is not possible based on appearance either because morphological differences overlap or because of morphological variability. For example, the earthworms *Lumbricus terrestris* and *Lumbricus herculeus* appear to differ in segment number, body mass, and body length but identification is not reliable because their measurements overlap (James et al., 2010). Snails in the genus *Radix* cannot be distinguished based on shell morphology because it is variable, overlapping among species and is phenotypically plastic depending on environmental conditions (Pfenninger, Cordellier, & Streit, 2006). The problem is especially acute among embryonic and larval forms before the development of morphological characteristics that distinguish adults.

To distinguish morphologically similar species, DNA barcodes based on the sequence of the mitochondrial cytochrome oxidase subunit I (COI) gene (Hebert et al., 2003) are now widely used. The present study examines the utility of an alternative method to detect sequence variations for the same purpose. High‐resolution melting (HRM) analysis reveals sequence variation based on the melting temperature of PCR amplicons. The melting temperature is determined post‐PCR by monitoring the fluorescence of amplicons in relationship to temperature in the presence of a dsDNA saturating dye. Sequence variation is most obvious in short amplicons for which small changes can significantly affect the melting temperature. At approximately 700 base pairs, it was unclear whether differences in the melting temperature of the amplified fragment of the COI gene would be useful for distinguishing animal species.

The melting peak profiles of the COI amplicon appeared highly distinguishable among species for both *Lithobates* (Figure 1a) and *Hyla* (Figure 1b) suggesting that HRM analysis may be a useful way to distinguish related species. Different regions of the COI amplicon melted at different temperatures, forming melting domains that together created unique melting peak profiles. Sequence variation within a species shifted the melting temperature slightly of one or more melting domains but does not appear to affect the distinctiveness of the melting peak profiles. Greater sequence variation among species, however, does affect the distinctiveness of the melting peak profiles, demonstrating the usefulness of this method.

The melting profile of amplified DNA depends on sequence, length, GC content, and heterozygosity (Farrar & Wittwer, 2017; Reed, Kent & Wittwer, 2007; Steer et al., 2009) and the melting profiles of two species can be similar by happenstance. This was observed in the similarity between the COI melting profiles of the frogs *Lithobates sphenocespala* and *G. carolinensis*. The two frogs belong to different families (Ranidae and Microhylidae, respectively) with sequence differences in the COI barcode up to 145 nt between individuals of the two families (data not shown) yet have similar COI melting profiles. On the other hand, congeners of both *Lithobates* and *Hyla* frogs have distinguishable COI melting profiles. This highlights the point that COI melting profiles are simply an easy way to reveal COI sequence differences but convey no phylogenetic information.

We think HRM analysis can be useful in two ways, either to distinguish morphologically similar species or to confirm species identity. To distinguish morphologically similar species when some discriminatory criteria are available (e.g., eye and spiracle position in larval anurans), melting peak profiles can be used to confirm species distinction as performed in the present study. For species confirmation, melting profiles of samples can be compared to those of reference species in a database. Alternatively, sample DNA (e.g., from a juvenile for which morphological characteristics are ambiguous) can be spiked with an equivalent amount of reference DNA (e.g., from an adult of certain identity) before performing PCR‐HRM. If both samples can be amplified with similar efficiency, a single melting profile would confirm species identity. This later approach was used successfully in this study to confirm the identity of *L. catesbeianus* and *L. sphenocephala* tadpoles (data not shown).

In conclusion, we used anurans to show that HRM analysis of the COI barcode can be used to distinguish related species. COI‐HRM analysis offers no advantage over traditional sequencing. However, it is much faster because there is no need to do sequencing. It is a closed‐tube method by which the PCR and HRM steps are performed in the same reaction tube thereby eliminating the need for any post‐PCR manipulations. Although the cost of DNA sequencing is now relatively low, it can be a barrier for laboratories that are not well funded or for laboratories with large numbers of samples to study. After DNA extraction, the analysis only takes a few hours to complete and species differentiation relies only on visual comparison of the melting peak profiles. The only requirement is that the user has access to a thermal cycler capable of real‐time fluorescence measurements.

## CONFLICT OF INTEREST

None declared.

## AUTHOR CONTRIBUTIONS

S.E. and S.Y.W. conceived the study. S.E. obtained specimens and performed the DNA extractions and high‐resolution melting analysis of amplicons. S.E. and S.Y.W. wrote and approved the final manuscript.

## Data Availability

PCR amplicon sequences are available via GenBank (ascension numbers KT388385–KT388408).
